# Recent advancements in implantable neural links based on organic synaptic transistors

**DOI:** 10.1002/EXP.20220150

**Published:** 2023-11-07

**Authors:** Swarup Biswas, Hyo‐won Jang, Yongju Lee, Hyojeong Choi, Yoon Kim, Hyeok Kim, Yangzhi Zhu

**Affiliations:** ^1^ School of Electrical and Computer Engineering, Center for Smart Sensor System of Seoul (CS4) University of Seoul Seoul Republic of Korea; ^2^ Terasaki Institute for Biomedical Innovation Los Angeles California USA; ^3^ Central Business, SENSOMEDI Cheongju‐si Republic of Korea; ^4^ Institute of Sensor System, SENSOMEDI Cheongju Republic of Korea; ^5^ Energy Flex Seoul Republic of Korea

**Keywords:** artificial synapses, flexible electronics, implantable synaptic transistor, neural link, organic transistor

## Abstract

The progress of brain synaptic devices has witnessed an era of rapid and explosive growth. Because of their integrated storage, excellent plasticity and parallel computing, and system information processing abilities, various field effect transistors have been used to replicate the synapses of a human brain. Organic semiconductors are characterized by simplicity of processing, mechanical flexibility, low cost, biocompatibility, and flexibility, making them the most promising materials for implanted brain synaptic bioelectronics. Despite being used in numerous intelligent integrated circuits and implantable neural linkages with multiple terminals, organic synaptic transistors still face many obstacles that must be overcome to advance their development. A comprehensive review would be an excellent tool in this respect. Therefore, the latest advancements in implantable neural links based on organic synaptic transistors are outlined. First, the distinction between conventional and synaptic transistors are highlighted. Next, the existing implanted organic synaptic transistors and their applicability to the brain as a neural link are summarized. Finally, the potential research directions are discussed.

## Graphical abstract

Rapid advancement in brain synaptic devices uses field effect transistors to simulate human brain synapses. Organic semiconductors, which are noted for their simplicity and biocompatibility, have enormous potential in brain synaptic bioelectronics. This review discusses ongoing obstacles, even with employment in integrated circuits and brain networks, and covers recent improvements, their application, and future research prospects.

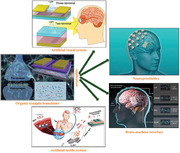

## 1 INTRODUCTION

The human brain contains 10^11^ neurons that operate on minimal power, have a high fault tolerance, and can perform effective parallel processing. Motivated by these distinguishing characteristics, scientists have endeavoured to develop artificial synapses that raise inspiration from the architecture and functionality of the human brain, which has evolved to perform intricate cognitive tasks.^[^ ^1^ ^]^ Synaptic electronics are projected to reign in an entirely novel age of bioinspired electronics in next‐generation smart neurorobotics.^[^ ^2^ ^]^ A variety of current synaptic devices exhibit capabilities in areas of synaptic bionics and neural state processing. These neuronal devices can be classified into two categories: (1) dual‐terminal devices, encompassing memristors, atomic switches, and ferroelectric devices; and (2) tri‐terminal transistors, including ferroelectric gate field‐effect transistors, electrolyte‐gated transistors, floating‐gate transistors, and nanoparticle transistors.^[^ ^3^, ^4^, ^5^, ^6^, ^7^, ^8^, ^9^, ^10^, ^11^, ^12^ ^]^ Synaptic transistors based on organic semiconductors (OSCs) represent promising components for developing novel flexible electronics. Their utility lies in their ability to enable the fabrication of large‐scale electronic devices using additive manufacturing techniques, such as inkjet printing. This paves the way for advanced and flexible electronic systems.^[^ ^13^ ^]^ Organic synaptic transistors are promising candidates for building implantable and wearable electronics when compared to other material‐based synaptic transistors.

Like that, wearable electronic skin can be developed via an organic synapse that continually works under folding conditions on uneven surfaces.^[^ ^14^, ^15^, ^16^, ^17^, ^18^ ^]^ Additionally, organic synaptic transistors are biocompatible, allowing researchers to bridge the gap between electronics and biology by recording neuron action potentials.^[^ ^19^, ^20^ ^]^ In order to improve performance and eliminate intercellular cross‐talk, such transistors have evolved from two‐terminal to three‐terminal devices in recent years. Vertically organized three‐terminal devices can form a highly linked neural network system, augmenting their functionality.^[^ ^21^, ^22^, ^23^ ^]^ Moreover, the presynaptic input signal cannot easily disrupt the postsynaptic received signal in three‐terminal devices, simplifying the construction of multi‐input devices.^[^ ^24^ ^]^ In addition to their unique characteristics, such as electrical functionality that may be altered by molecular engineering, cost‐effective manufacturing processes, favourable mechanical properties, low energy consumption, and biocompatibility, organic synaptic transistors have emerged as viable elements for bio‐hybrid neuromorphic systems.^[^ ^25^, ^26^ ^]^ Organic synaptic transistors, which employ electrolytes as gate dielectric, can process biological information such as electrophysiological, biophysical, and biochemical signals. Their unique characteristic enables seamless integration of these devices with biological entities and organs, facilitating applications such as implantable neural links. In the future, organic synaptic transistors may substitute biological nerves partly or entirely as implantable neural links to treat neural disorders.^[^ ^27^, ^28^ ^]^ For such biomedical applications, bio‐signals should be precisely monitored, and it is expected to substitute biological nerves while minimizing any negative impacts on the body. Many scientists have developed safe and functionally integrated bio‐hybrid neuromorphic systems and gadgets based on organic synaptic transistors.^[^ ^29^, ^30^ ^]^ Therefore, a thorough review is essential to promote the use of such transistors in the abovementioned applications.

Considering these aspects, we discuss the recent developments in implantable neural links based on organic synaptic transistors. First, we highlight the differences between conventional and synaptic transistors. Next, we introduce the different types of implantable organic synaptic transistors and their application to the brain as a neural link. Finally, we discuss the future outlook and potential research directions.

## 2 SYNAPTIC TRANSISTORS

### 2.1 Differences between conventional and synaptic transistors

The human nervous system serves as an electrical conduit to transmit external stimuli to the brain, where a comprehensive evaluation takes place to determine an appropriate response, drawing upon stored experiences from memory. Subsequently, the decision outcome instructs the motor system to carry out a responsive action in the body.

**FIGURE 1:**
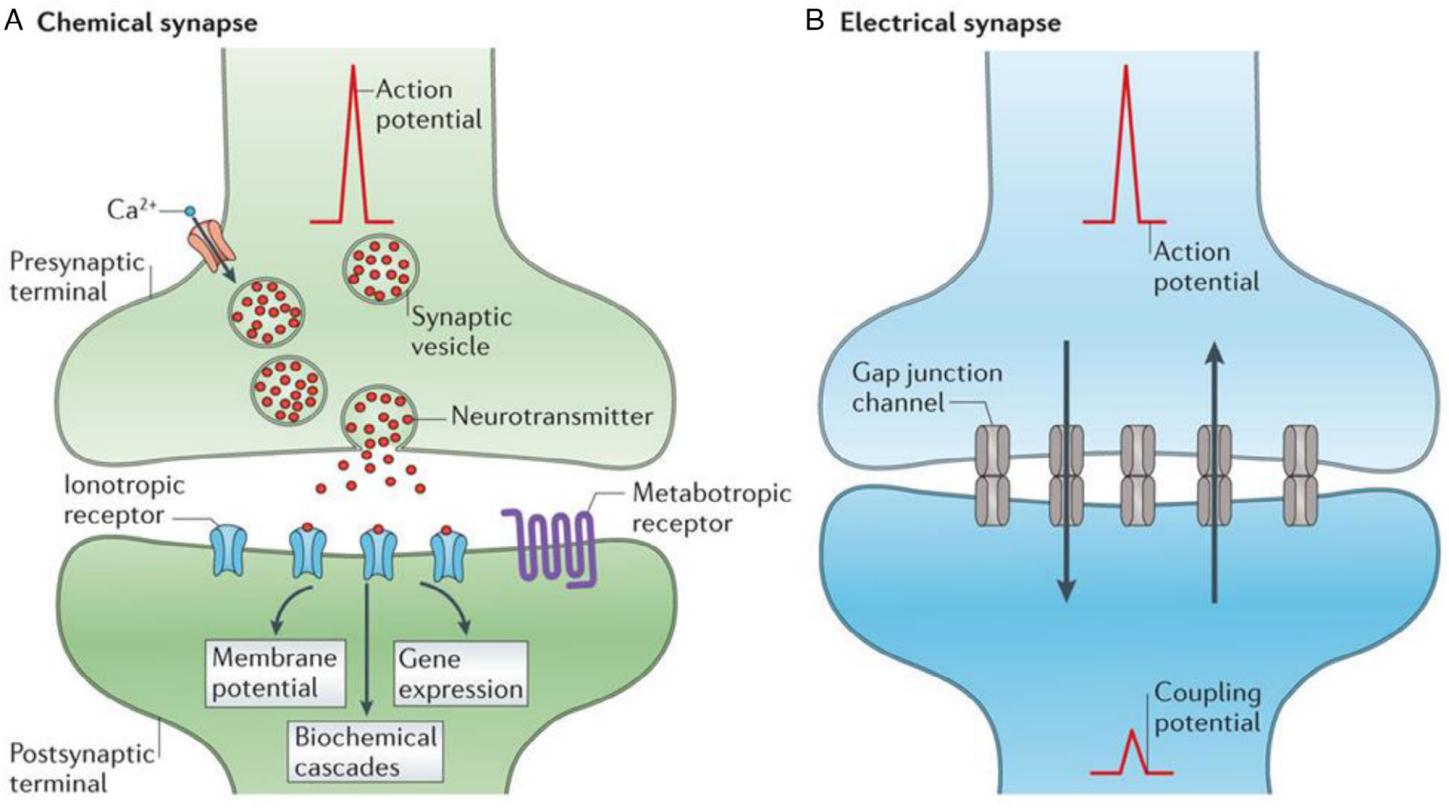
Schematic illustration of a biological synapse. There are two types of synapses: chemical synapses and electrical synapses. Chemical synapses transmit signals through the release and diffusion of neurotransmitters. On the other hand, electrical synapses facilitate rapid communication between cells by utilizing direct electrical coupling through gap junctions. Reproduced with permission.^[^ ^31^ ^]^ Copyright 2014, Springer Nature Publishing AG.

The existence of neurons, specialized cells that send messages quickly and accurately to other cells, characterizes the nervous system at the cellular level. These cells are equipped with unique structures, including thin fibres named axons, which facilitate the transmission of electrochemical impulses. Depending on the type of synapse, signals can be transmitted directly between neighbouring cells through electrical synapses or via chemical messengers known as neurotransmitters released at chemical synapses^[^ ^31^ ^]^ (Figure 1).

Electrical synapses, which conduct signals faster than chemical synapses, are typically used in neural systems that necessitate an instantaneous response, such as defensive reflexes. However, unlike chemical synapses, electrical synapses lack gain, resulting in a postsynaptic signal that is either identical to or weaker than the presynaptic signal. Chemical synapses, which can exhibit a gain of 1 or more and alter the sign of the bio‐potential, play a vital role in the biological computations underlying perception and cognition, in contrast to electrical synapses. As a result, the majority of artificial synaptic devices are created to resemble chemical synapses. Hence this review focuses on chemical synapses.

A neuron receives signals through its dendrites and transmits them to the cell body. A spike is generated if the signals there surpass a particular threshold after being combined spatially and temporally. Once developed, an action potential propagates along the axon and releases neurotransmitters from synaptic vesicles into the synapses upon reaching the axon terminal. Many of these neurotransmitters are intricately linked to ionotropic receptors situated on the membrane of the postsynaptic terminal. Depending on the type and quantity of neurotransmitters released, this binding event can alter the magnitude and polarity of the signal potential of the postsynaptic neuron. Excitatory neurotransmitters (e.g., glutamatergic agents) generate depolarizing excitatory postsynaptic potentials (EPSPs) corresponding to a positive voltage.

**FIGURE 2:**
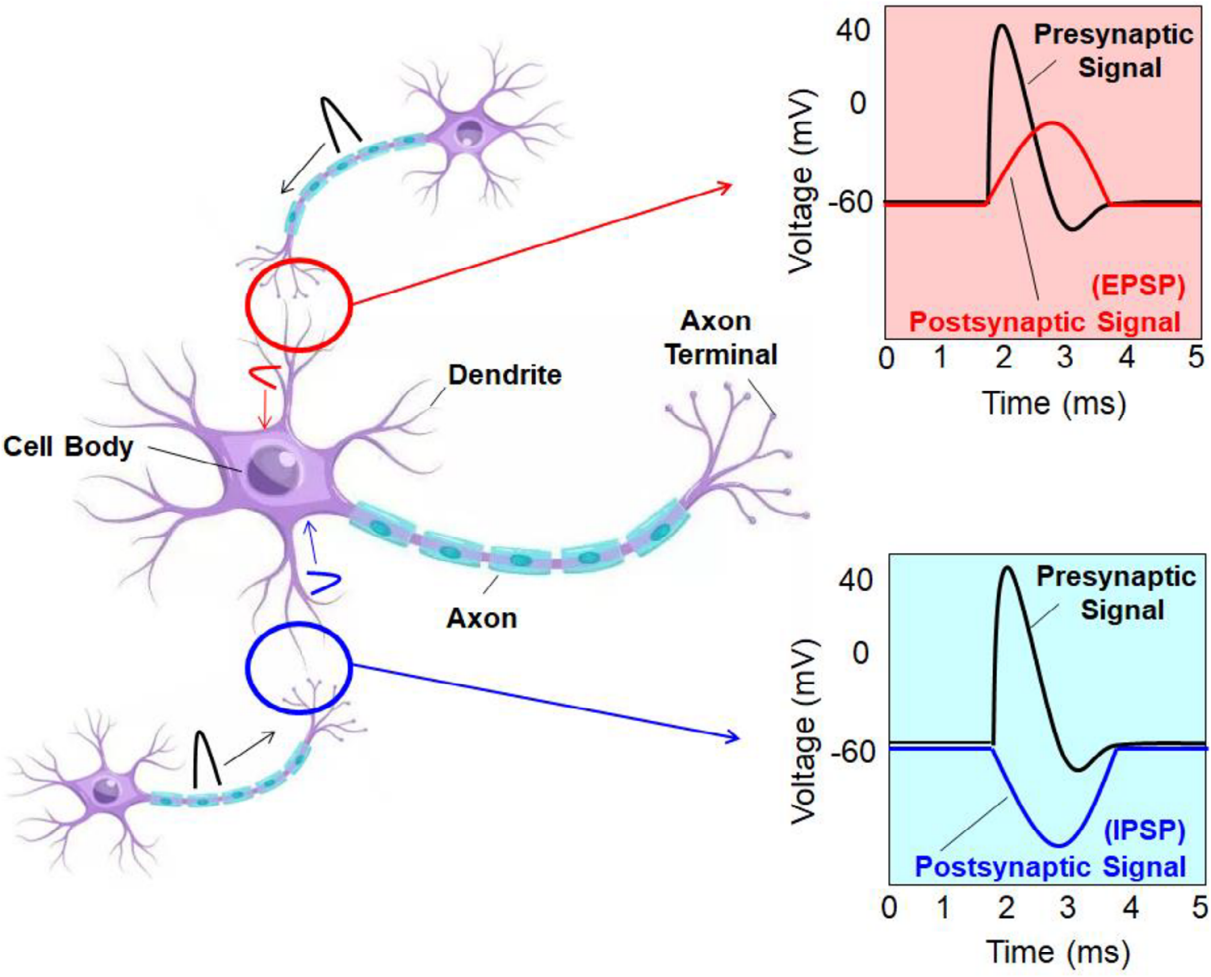
Schematic diagram of neurons. Excitatory postsynaptic potentials (EPSPs) and inhibitory postsynaptic potentials (IPSPs) are two types of electrical changes that occur in the postsynaptic neuron in response to neurotransmitter binding at a synapse. EPSPs are depolarizing electrical changes in the postsynaptic neuron's membrane potential. IPSPs, on the other hand, are hyperpolarizing electrical changes in the postsynaptic neuron's membrane potential.

In contrast, inhibitory neurotransmitters (e.g., GABAergic agents) cause hyperpolarizing inhibitory postsynaptic potentials (IPSPs) corresponding to a negative voltage (Figure 2). The vital function of synapses is to regulate the signal transmitted from the presynaptic neurons to the postsynaptic neurons. Presynaptic signal and synaptic weight may be combined to describe this function, and ordinary transistors, which are often used as voltage‐controlled current sources in electronic systems, can be used to implement it. However, biological synapses have another critical feature beyond simple signal regulation: synaptic plasticity.

When a new stimulus enters our brain, the synaptic vesicles and receptors are not abundant; thus, neurotransmitters are transmitted less efficiently. On the other hand, the synaptic structure adjusts in response to repeated exposure to the same stimuli, resulting in an increase in synaptic vesicles and receptors and an improvement in the transit of neurotransmitters. This change is sustained for a certain time (retention time). This phenomenon is termed synaptic plasticity, which was first proposed by Donald O. Hebb as Hebbian learning in 1949.^[^ ^32^ ^]^ The academic community accepts the hypothesis that synaptic structural changes could be a phenomenon of learning and memory based on Hebbian learning, which states that “neurons that fire together, wire together.” Signals are produced when people encounter or learn something, and the connections between the synapses associated with those signals become stronger and increase in number, resulting in the formation of long‐term memories in the brain.

Based on retention duration, synaptic plasticity may be classified into two groups: short‐term plasticity (STP) and long‐term plasticity (LTP).^[^ ^33^ ^]^ Within milliseconds to minutes, STP takes place and is in charge of crucial computational operations in neural networks. Contrarily, LTP entails modifications that endure for several hours or longer, resulting in long‐lasting changes to the neural network that enable the brain to store vast amounts of information. Biological synapses serve a variety of STP and LTP‐related purposes. Paired‐pulse facilitation (PPF), which occurs when the following peak comes after the preceding rise, is one of the functions of STP.

Similarly, enduring external stimulation can modify synaptic architecture, leading to a shift from STP to LTP, establishing the physiological basis for memory formation and learning. In line with Hebb's hypothesis, spike‐time‐dependent synaptic plasticity (STDP) suggests that the timing relationship between presynaptic and postsynaptic spikes can influence the effectiveness (synaptic weight) of synaptic transmission.^[^ ^31^ ^]^ When a presynaptic neuron spikes before a postsynaptic neuron, the synaptic connection is strengthened, and the synaptic weight increases. However, the synaptic weight decreases if the postsynaptic neuron spikes first, followed by the presynaptic neuron. Smaller spike‐time disparities result in more significant weight shifts, which is a crucial characteristic. In addition to STDP, a fundamental process known as spike‐rate‐dependent synaptic plasticity (SRDP) modifies synaptic weight by regulating the rate at which presynaptic spikes fire. For instance, low‐frequency (1–5 Hz) and high‐frequency (20–100 Hz) trains, respectively, lead to long‐term depression and long‐term potentiation.

Memory functions and the signal modulation capability of conventional transistors are required to implement such synaptic plasticity. Several memory techniques, such as charge‐trap flash memory, resistive random access memory, spin‐transfer torque magnetic random access memory, phase change memory, and static random access memory, have been suggested as potential options for integrating synaptic transistors.^[^ ^34^ ^]^ Many existing neural systems have been based on implementing synaptic plasticity behaviours in synaptic transistors, including EPSP, IPSP, PPF, STDP, and SRDP.

Notably, synaptic and conventional transistors have different requirements. Typically, conventional transistors require fast operating speeds of nanoseconds and significant on‐current characteristics to implement digital systems operating at hundreds of megahertz or more. In contrast, the human nervous system operates at speeds of 1–10 Hz, and thus, synaptic transistors require operating speeds of the order of micro‐ to milliseconds and on‐current characteristics that are 1/10 to 1/100 of those of conventional transistors. Moreover, different photosensitive transistors at different wavelengths are indispensable for intelligent perception.^[^ ^35^, ^36^, ^37^ ^]^ In other words, the drawback of lesser mobility compared to conventional silicon‐based transistors may be successfully solved by introducing synaptic transistors made of biocompatible organic materials. The differences between traditional and synaptic transistors, including the mentioned aspects, are summarized in Table 1.

**TABLE 1:** The differences between conventional transistors and synaptic transistors.

	Conventional transistor	Synaptic transistor
Target operation	Analog/digital signal control	Biomimetic synapse operation (synaptic plasticity)
Operation principle	Current modulation	Resistance (or conductance) modulation
Memory function	Not required	Required
Operation speed	>1MHz	<100 Hz
Current characteristic	Large on‐current characteristic is crucial for achieving fast speed.	Small on‐current characteristic is important for achieving low energy consumption.
Main material	Rigid and heat‐resistant materials.	Flexible and biocompatible materials
Application	Conventional electronic system (i.e., smartphone)	Brain‐like electrical system (i.e., neurorobotics, E‐skin)

### 2.2 Implantable synaptic transistors

#### 2.2.1 Organic transistors

**FIGURE 3:**
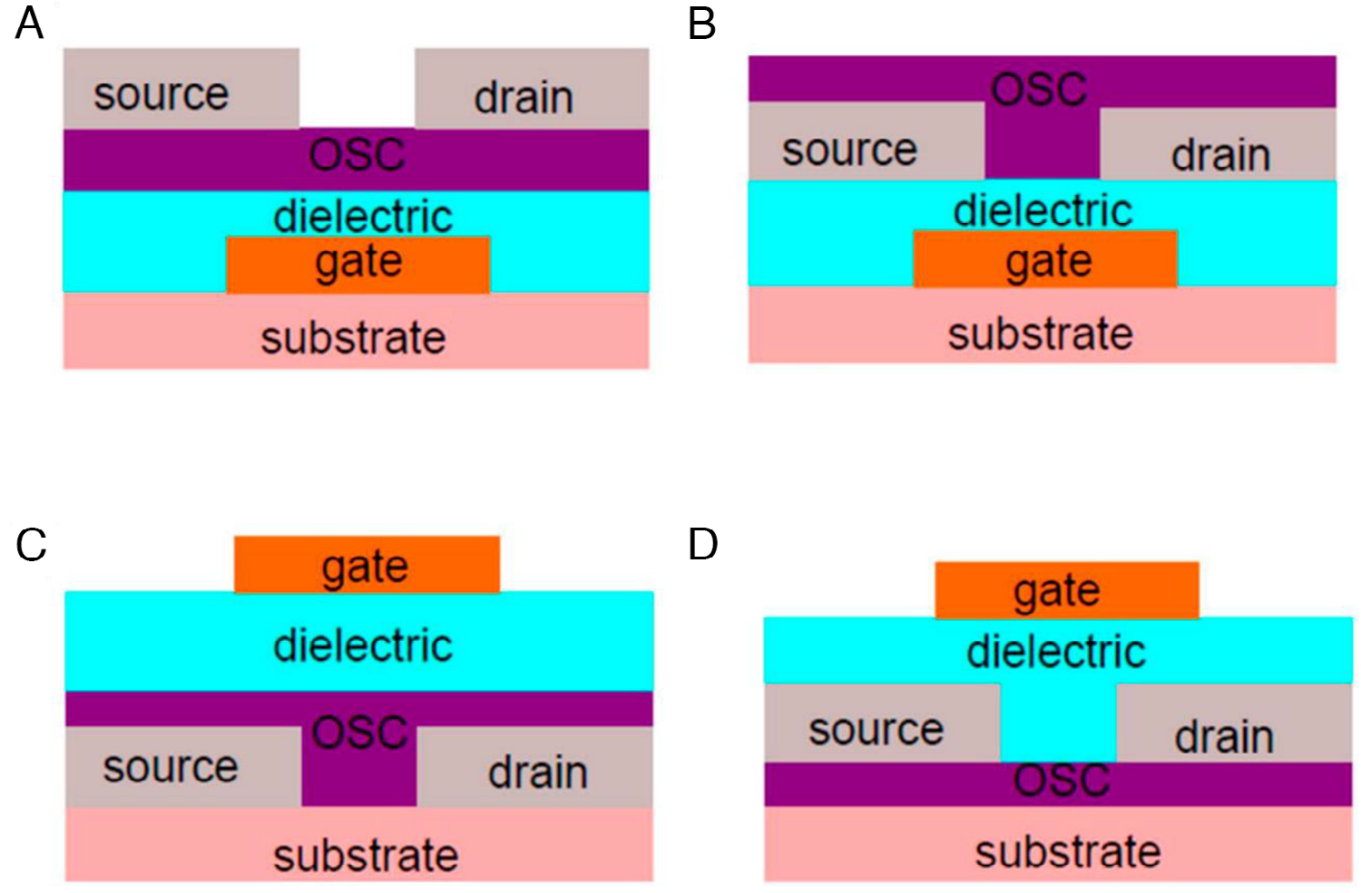
Different geometrical configurations of an OFET. (A) Bottom gate, top contact (BGTC), (B) bottom gate, bottom contact (BGBC), (C) top gate, bottom contact (TGBC), (D) top gate, top contact (TGTC) OFET.

Organic field effect transistors (OFETs), known as organic thin‐film transistors, are three‐electrode devices. Because of its numerous advantages, such as low‐temperature processing, super mechanical flexibility, and compatibility with low‐cost and high throughput manufacturing, OFETs are employed in combination with a wide range of electronic devices, from chemical sensors for volatile organic compounds to near‐infrared‐sensitive phototransistors and biosensors.^[^ ^16^, ^38^ ^]^ OFET devices are renowned for their mechanical flexibility, low processing temperature, and chemical utility. Furthermore, a variety of substrate substance options are available. For example, users can use paper, textiles, or other biodegradable substrates to reduce electrical trash. An OFET device is made up of three parts: The OSC, which serves as the active layer, is one component. The second component is the insulating layer, which has a capacitive function. A dielectric coating with a high dielectric constant is necessary to run OFETs at low voltages. The electrodes, which are designated gate (G), source (S), and drain (D), constitute the third component. By altering the electrode positions, many device designs may be created, with popular configurations being the bottom gate/top contact (BG/TC) (Figure 3A), bottom gate/bottom contact (BG/BC) (Figure 3B), top gate/bottom contact (TG/BC) (Figure 3C), and top gate/top contact (TG/TC) (Figure 3D). OFETs with the same components do not behave similarly with different device configurations. The insulator layer blocks charge carriers, which causes them to start accumulating. Therefore, the OFET operates under an accumulation regime. The channel conductivity is modulated by the gate electrode. The charge carriers are pumped and extracted by the source and discharge electrodes, respectively. When no voltage is supplied to the gate electrode (*V_G_* = 0), the “off‐state” occurs when very little current flows across the channel. When a gate voltage is applied, charge carriers congregate at the semiconductor/dielectric interface, forming a conductive active channel between the source and drain. The device switches to the “on‐state” as the presence of a mobile carrier at the interface results in an amplification of the current magnitude.

The primary parts of OFETs are universally acknowledged to be OSCs. Intermolecular π‐orbital overlaps and the chemical topologies of the conjugated frameworks significantly impact OFET carrier motion.^[^ ^39^ ^]^ OSCs may be divided into three groups: conjugated polymers, oligomers, and organic molecules. Because of their poor electrical characteristics and processing and purification challenges, oligomers are rarely explored. In comparison, organic molecules and conjugated polymers have been widely employed in flexible electronic devices due to their exceptional bendability, enhanced molecular packing efficiency, economical nature, adaptability to low‐temperature manufacturing, and superb compatibility with flexible substrates.^[^ ^40^, ^41^ ^]^ Owing to the presence of few grain boundaries, defects, and traps, the mobility of organic molecules is comparable to that of polycrystalline silicon FETs (>10 cm^2^ V^‐1^ s^‐1^), such as 2,9‐didecyl‐dinaphtho[2,3‐b:20,30‐f]thieno[3,2‐b]thiophene (C10‐DNTT, 10.7 cm^2^ V^‐1^ s^‐1^), rubrene (24.5 cm^2^ V^‐1^ s^‐1^), and pentacene, 2,7‐dioctyl benzothieno[3,2‐b] benzothiophene (C8‐BTBT, 31.3 cm^2^ V^‐1^ s^‐1^).^[^ ^42^, ^43^, ^44^ ^]^ In comparison with polymer semiconductors, organic small molecules have a larger molecular weight, a more regulated molecular structure, more mechanical flexibility, and a better capacity to assemble films.^[^ ^45^ ^]^ The highly structured lamellar structure and high crystallinity of polymer semiconductors also help to promote carrier motion. Still, they also limit mechanical stretchability, which is often facilitated by an amorphous form.^[^ ^46^ ^]^ Consider the side‐chain‐rich poly(3‐hexylthiophene) (P3HT), which features edge‐on stacking along the substrate and a lamellar packing arrangement of conjugated backbones. In comparison, poly‐(2,5‐bis(3‐alkylthiophene‐2‐yl)thieno [3,2‐b]thiophene], characterized by a more significant number of well‐defined crystalline domains with larger crystals and reduced imperfections, exhibits enhanced capability in producing highly organized films.^[^ ^47^, ^48^ ^]^

Appropriate dielectric materials must be selected to develop high‐performance OFETs. Traditional solid oxides like SiO_2_ are excessively rigid and fragile to be utilized as flexible dielectric materials. Polymer dielectrics, on the other hand, have several intrinsic advantages, including low‐temperature solution processing, low film density, significant leakage current density, outstanding compatibility with flexible substrates, adjustable molecule shapes, and molecular weight.^[^ ^49^ ^]^ Currently, poly (methyl methacrylate), polystyrene (PS), and poly (‐methylstyrene) are three of the flexible polymer dielectrics that are frequently used. Poly(dimethylsiloxane) (PDMS) and polystyrene‐block‐poly(ethylene‐ran‐butylene)‐block‐polystyrene are two commonly utilized stretchy polymer dielectrics.^[^ ^50^, ^51^, ^52^, ^53^, ^54^, ^55^ ^]^ Particularly, the dielectric constants (*k* = 3.0) found in these polymer rigid dielectric materials are less compared to those of inorganic rigid dielectrics, which may raise the operating voltage of OFETs. Therefore, the need to guarantee minimal energy requirements for wearable electrical gadgets cannot be met by these devices. By using conducting fillers and metal particles, it is possible to successfully raise the dielectric constant of polymer dielectrics.^[^ ^56^, ^57^, ^58^ ^]^ A standard solution to this issue is the cross‐linking of polymer dielectrics.^[^ ^59^ ^]^

The electrode type, which has drawn much research interest, is another important factor in the design of OFETs. To achieve the production of OFETs with outstanding performance, it is crucial to employ electrodes that exhibit excellent flexibility, transparency, conductivity, appropriate work function, minimal semiconductor contact resistance, and suitable biocompatibility. Since a few decades ago, optoelectronics has extensively used indium tin oxide (ITO) sheets, which are both optically transparent and electrically conductive. ITO films, on the other hand, are hard and brittle, and they rapidly fracture under minute tensile strains. Due to their superior conductivity and transparency, metal sheets of Au, Ag, and Al are often employed as electrode materials. In response to the fast growth of wearable electronics, several flexible electrodes, such as metal nanoparticles (NPs), metal nanowires, conductive polymers, carbon‐based nanomaterials, and ionic conductors, have been invented.^[^ ^60^, ^61^, ^62^, ^63^, ^64^, ^65^ ^]^

All OFET components are built on top of substrates. Flexible substrates have been made from thin glass, metal foil, polymers (plastics and elastomers), and a few unusual materials, including paper and fibre. Like polymer dielectrics, polymer surfaces offer several inherent advantages, including robust temperature resilience and resistance to corrosive fluids. Due to their potential use as artificial synapses (as implanted brain linkages), organic synaptic transistors have drawn the most scientific attention among the many types of OFETs. In the following section, we describe the recent advancements in organic synaptic transistors.

#### 2.2.2 Organic synaptic transistors

**FIGURE 4:**
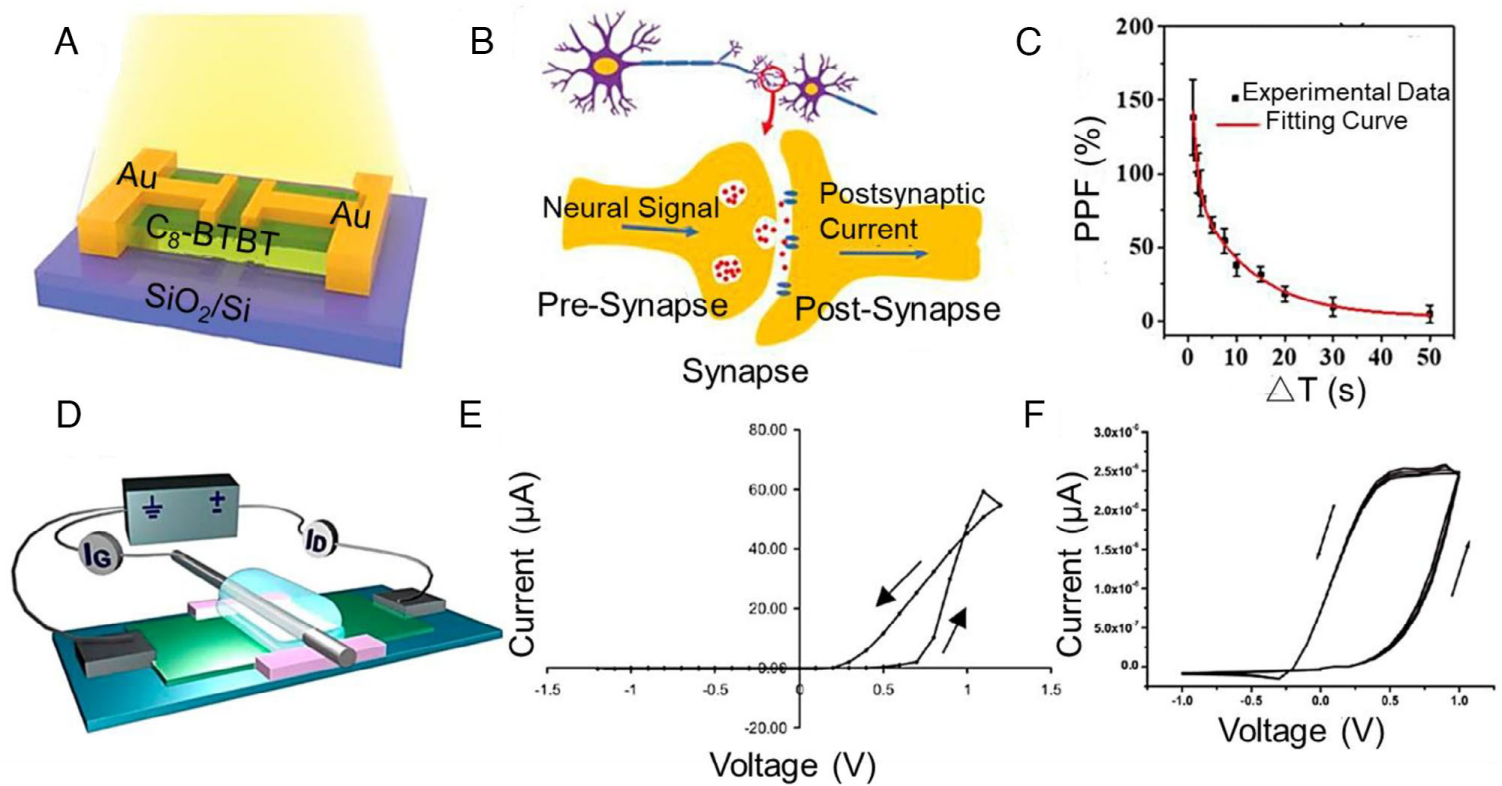
Organic synaptic transistors. (A) Schematic of the 2D thin film phototransistor, (B) schematic of nerve cells and signal transmission in the biological synapse, (C) standard errors from three independent tests of a synaptic transistor. Reproduced with permission.^[^ ^68^ ^]^ Copyright 2019, Wiley; (D) Schematic of the organic memristor with external power supply and measurement devices, (E) cyclic voltage–current characteristics of the organic memristor (The arrows indicate the direction (increase or decrease) of the voltage scan), (F) cyclic voltage–current characteristics for a sandwich structure in the fifth, sixth, and seventh cycles (The arrows indicate the direction (increase or decrease) of the voltage scan). Reproduced with permission.^[^ ^82^ ^]^ Copyright 2009, American Institute of Physics.

Newly developed synaptic modelling methodologies use three‐ and multiterminal transistors to accomplish proper device selections via the gate voltage while preventing the problem of neighbouring cell coupling exhibited in two‐terminal synaptic electronic devices. The electrical signal produced by the presynaptic terminal of the gate electrode is employed to control the postsynaptic terminal of organic synaptic transistors. The postsynaptic terminal, comprising both the source and drain electrodes, is situated within the active layer. It is believed that the altered postsynaptic terminal represents an alteration in synaptic weight, which affects the capacity of organic synaptic transistors to hold charges. Numerous neural functions can be imitated by various organic synaptic transistor materials and architectures.^[^ ^9^, ^12^, ^66^ ^]^ This section describes the components, structures, and operating mechanisms of organic synaptic transistors. Notably, such transistors can facilitate the formation of an organic synapse cell. Diverse semiconductor materials can be used to create organic synaptic transistors, which may imitate neurons in various ways. For instance, devices constructed using light‐sensitive OSC materials (such as C8‐BTBT, perovskite quantum dots (QDs)/conjugated polymers, single crystals of 2,6‐dithienylanthracene (DTAnt), and organic molecular crystals) frequently mimic visual neurons, allowing for the simulation of functionalities resembling radio transmission and digital recognition.^[^ ^67^, ^68^, ^69^, ^70^, ^71^, ^72^, ^73^ ^]^ The light that the photosensitive OSC creates while operation alters the carrier in the channel, which changes the current signal between the source and drain electrodes. Also, the interfacial charge entrapment effect explains the memory characteristics of developed synaptic devices. It has been discovered, for example, that a specific 2D C8‐BTBT OFET with simple solution epitaxy at room temperature may imitate a variety of fundamental, typical synaptic processes (Figure 4A–C).^[^ ^68^ ^]^ The active channel layer of an anion‐doped polymer semiconductor has also been shown to imitate synaptic activity and enhance memory performance.^[^ ^74^ ^]^ Poly(3,4‐ethylene‐dioxythiophene): poly(styrene sulfonate) and poly(3,4‐ethylene‐dioxythiophene): poly(styrene sulfonate) have been utilized as materials for producing organic electrochemical transistor components that exhibit synaptic functionalities.^[^ ^10^, ^75^, ^76^ ^]^ Additionally, organic synaptic transistors can be created by introducing metal NPs at the dielectric/OSC junction. The memory effect brought on by charge entrapment and de‐trapping may be modified with the aid of the NPs.^[^ ^77^, ^78^, ^79^ ^]^

The device platform is infused with an electrolyte until it contacts the gate electrode, creating a liquid ion electrolyte‐gate transistor configuration.^[^ ^80^ ^]^ Particularly, when employing liquid electrolyte‐gate transistors (using KCl and NaCl), the introduction of electrolytes into the PDMS well can impede the participation of atmospheric oxygen in the electrochemical reactions. The transfer of charge in the OSC and ionic coupling in the electrolyte solution must happen for the device to function.^[^ ^81^ ^]^ Using a liquid electrolyte gate, Erokhin and colleagues produced the initial organic synapses in 2009, and they also published the findings of an electrochemical gated polymer memory test^[^ ^82^ ^]^ (Figure 4D–F). Malliaras and colleagues report a liquid ion electrolyte‐gate transistor based on conductive PEDOT: PTHF that exhibits non‐volatile characteristics when the polymer structure undergoes a change in conformation under an elevated reduction potential.^[^ ^75^ ^]^ Synaptic integration, short‐term memory to long‐term memory transitions, remembering, and integrated information. Nonetheless, effectively controlling the flow of electrolytes and integrating them into pliable, adaptable transistors and circuits poses a challenge. Immobilizing ionic liquids in polymers by constructing a 3D network structure is a potential approach. Solid ion electrolytes have more application possibilities, flexibility, higher safety, and less leakage than liquid electrolytes. Electronic technology presents a vast array of potential applications due to the capability of solid‐state electrolytes to form a robust electrolytic double layer (EDL) with gate‐like properties and operate at low voltages.^[^ ^83^, ^84^ ^]^ Due to its nonvolatility, high on/off ratios, adaptable multilayer conductance states, and quick switching capabilities in OFETs, organic ferroelectric materials are also a preferred dielectric layer material for artificial brain networks.^[^ ^8^, ^14^, ^85^ ^]^ Ferroelectric field effect transistors (FeFETs) have good memory properties due to leftover ferroelectric material polarization in the gate medium.^[^ ^9^, ^86^, ^87^ ^]^ The ferroelectric material generates spontaneous polarization in the context of an electric field that may be adjusted. Following the reduction of the external electric field, some spontaneous polarization remains and can be accumulated or anti‐polarized to maintain the channel switch state on the transistor channel surface. P(VDF‐TrFE), a ferroelectric polymer material that acquires a stable dipole moment due to the incorporation of fluorine and hydrogen ions with opposite charges into its primary chain, emerges as a highly promising option for utilization as a dielectric layer in FETs.^[^ ^88^, ^89^, ^90^ ^]^

In this section, we delve into two primary categories of organic synaptic transistors: floating‐gate transistors and EDL transistors (EDLTs). These transistors imitate the intrinsic capabilities of synapses observed in the brain. In a floating‐gate transistor, the control gate assumes a comparable function to the gate terminal in a conventional transistor, while the floating gate is situated between the obstructing and tunnelling dielectric layers.^[^ ^91^ ^]^ Floating gates frequently utilize metal NPs, organic/oxide NPs, organic/C60, QDs, proteins, and 2D materials.^[^ ^92^, ^93^, ^94^, ^95^, ^96^ ^]^ Charge carriers are easily able to enter the floating gate thanks to thermal emission or quantum tunnelling. The trapped charge carriers change the threshold voltage by blocking the longitudinal electric field between the control gate and the channel layer. Thus, floating‐gate trapping can be used to realize the current memory function. The erase method releases the floating gate's carriers by applying a voltage in the opposite direction to the programming orientation. No external gate voltage is needed to maintain the stored charge since the floating gate is surrounded by dielectric layers that block and tunnel.

**FIGURE 5:**
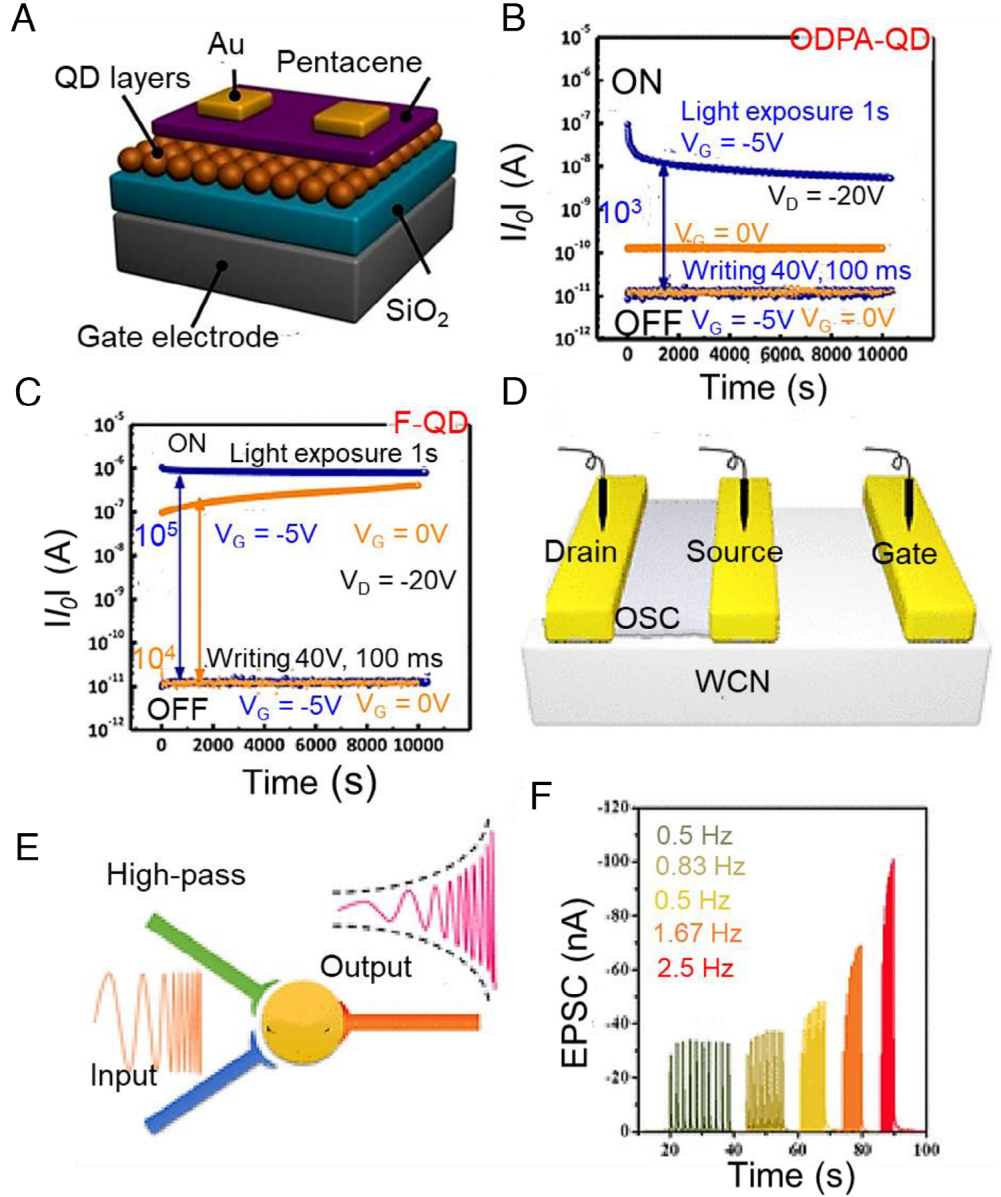
Organic synaptic transistors. (A) Schematic showing OFET incorporating the CdSe QD film, (B) retention test results of OFET memories with ODPA‐QD floating‐gate layers, (C) retention test results of OFET memories with F‐QD floating‐gate layers. Reproduced with permission.^[^ ^98^ ^]^ Copyright 2018, American Chemical Society; (D) schematic of laterally coupled C8‐BTBT field‐effect transistors, (E) schematic of a high‐pass filter in biological synapses, (F) EPSC signals in response to 10 presynaptic spike trains with different frequencies (The spike width and intensity were 200 ms and −1.5 V, respectively, the EPSC signals were recorded at a constant *V*_d_ of −0.1 V). Reproduced with permission.^[^ ^100^ ^]^ Copyright 2018, American Chemical Society.

Additionally, memory mode can make use of floating‐gate transistors.^[^ ^97^ ^]^ A visual synaptic device made of CdSe QDs with three distinct organic materials acting as drifting gates was created in 2018 by Yao's group^[^ ^98^ ^]^ (Figure 5A–C). Following that, the team of researchers introduced an innovative technique for fabricating self‐assembled floating gate organic transistors, distinguished by enhanced channel conductivity and memory functionalities influenced by activity‐dependent mechanisms. Finally, a floating‐gate design is advantageous for carrier storage and the fabrication of biological synaptic transistors.

Two layers of ion dispersion have been used to describe the EDL model: compact and diffuse layers.^[^ ^99^ ^]^ Strong ion adsorption occurs on the electrode surface of the compact layer, which is made up of an inner and an outer Helmholtz plane. The dielectric layer's key component is the solid ion electrolyte, and the electrostatic capacitive coupling effect is the EDL's primary functioning mechanism. Given their mode of operation, EDLTs may be transformed into multiterminal synaptic transistors and organic synaptic transistors with vertical architectures and many side gates.^[^ ^24^, ^100^, ^101^ ^]^ Many researchers have concentrated on developing side gate mechanisms in recent years. In instances involving multiterminal synapses or devices with vertical architectures, EDL configurations have been implemented utilizing various solid ion electrolyte materials, including chicken albumen, chitosan, ion‐gel, and cellulose nano‐papers derived from wood^[^ ^100^ ^]^ (Figure 5D–F). Qian and colleagues developed a multi‐gate organic neuron transistor specifically crafted for spatial information processing. This pioneering device incorporates P3HT as the active layer and utilizes ion glue as the insulating layer.^[^ ^102^ ^]^ The design of multi‐input coupling networks or other synaptic topologies that imitate the electrical signal processing function of brain networks has been a common use of EDLTs.

#### 2.2.3 Biomedical applications of implantable organic synaptic transistors

Implantable organic synaptic transistors represent a captivating field of research within the realm of biomedical applications. These devices can potentially revolutionize neuroprosthetics, brain–machine interfaces, and other neural implant technologies by emulating the intricate functionality of biological synapses in the human brain (Figure 1). Synapses facilitate the exchange of impulses between neurons, and depending on the transmission processes involved, they can be either electrical or chemical in nature. Electrical synapses utilize electrically connected gap junctions to rapidly transfer impulses, while chemical synapses involve the release of neurotransmitters into the synaptic cleft. These neurotransmitters diffuse and bind with receptors in postsynaptic cells, enabling the transmission of information from presynaptic to postsynaptic neurons.^[^ ^103^ ^]^ Presynaptic signals can be amplified and changed when neurotransmitters engage the receptors at chemical synapses, which can have complex consequences on the postsynaptic neurons. An EPSP or an IPSP can particularly be produced by presynaptic signals. The flexibility of synapses, which emerges as an activity‐dependent shift in the “weight” (or strength) of the connection between the presynaptic and postsynaptic neurons, is a crucial property of synapses. Synaptic plasticity helps to change thoughts, feelings, behavior, and memory.^[^ ^104^, ^105^, ^106^, ^107^ ^]^ Utilizing organic synaptic transistors to simulate STP and LTP in artificial sensory systems and neuromorphic computers has required much work. Analogous to biological synapses, ion‐gel‐gated organic synaptic transistors (IGOSTs) exhibit seamless transitions between STP and LTP.

**FIGURE 6:**
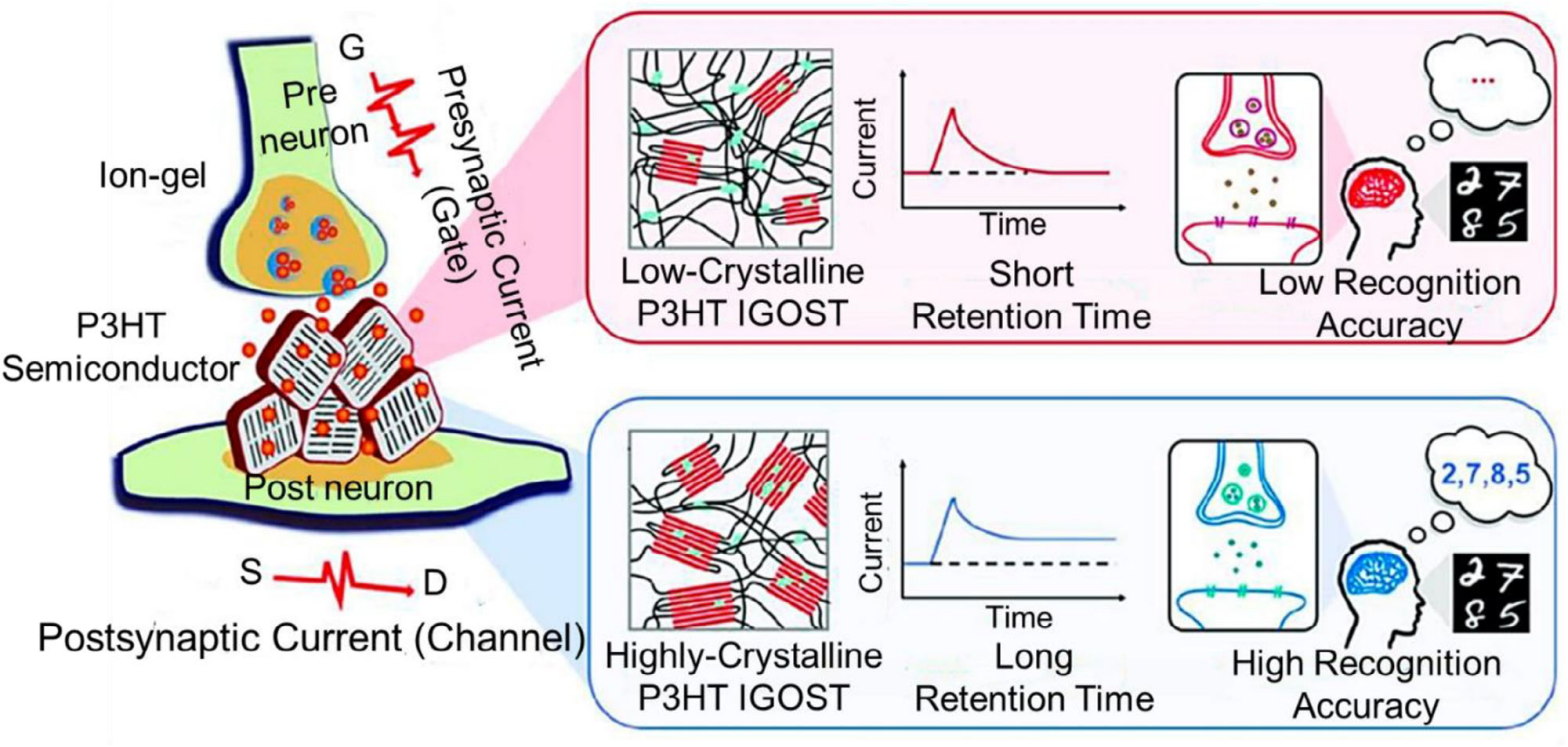
Biomedical Applications of Implantable Organic Synaptic Transistors. Schematic of IGOST (left) and microstructure‐controlled synaptic plasticity (right) in low‐crystalline P3HT IGOST (right, top) and highly crystalline P3HT IGOST (right, bottom). Change in the microstructure leads to change in the long‐term synaptic plasticity, which affects the recognition accuracy for neuromorphic computing. Reproduced under the terms of the Creative Commons Attribution 4.0 International license.^[^ ^108^ ^]^ Copyright 2020, The Authors. published by Wiley‐VCH GmbH.

**FIGURE 7:**
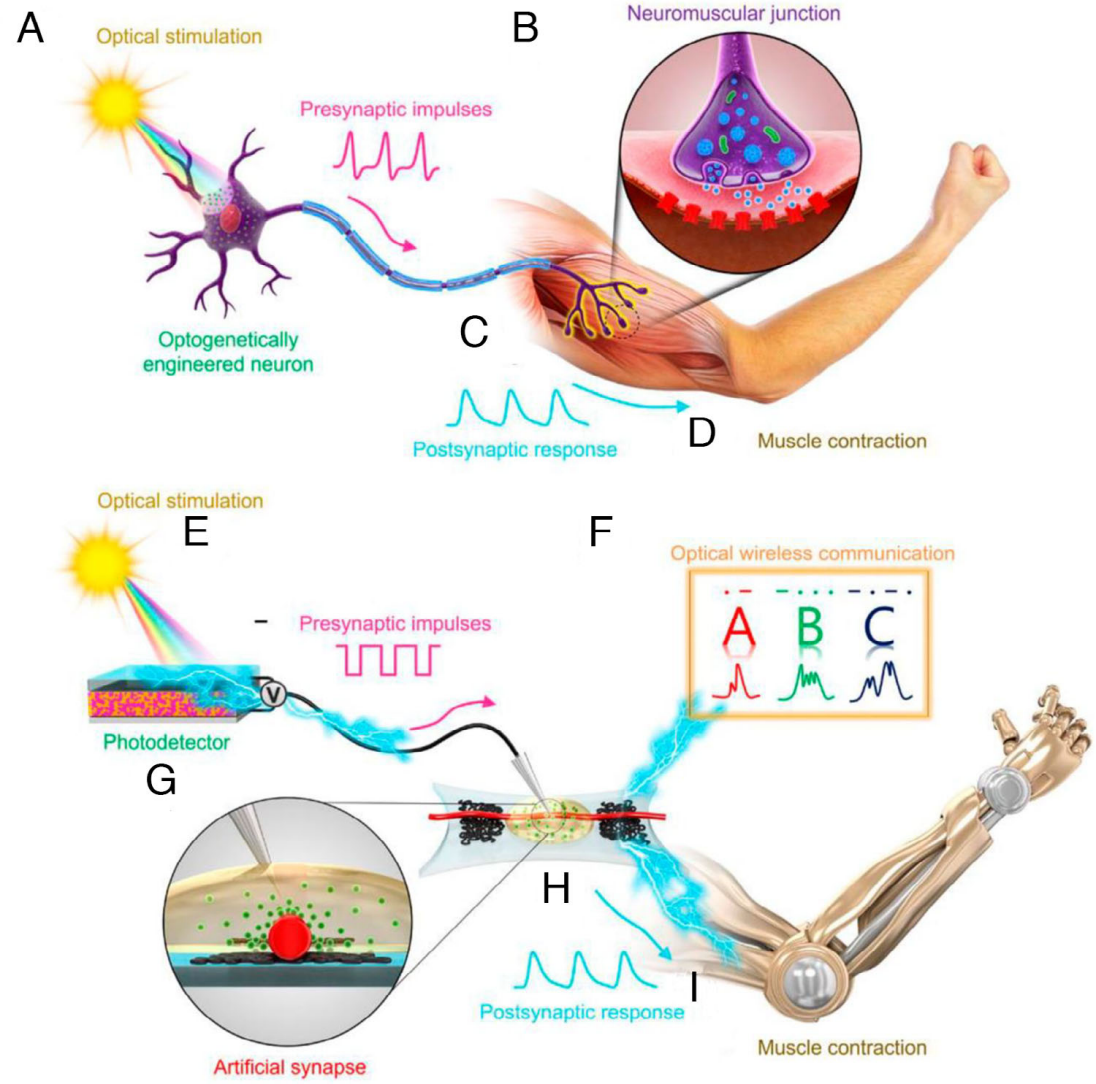
Biomedical applications of implantable organic synaptic transistors. Biological and organic optoelectronic synapse and neuromuscular electronic system. (A–D) In a biological system, (A) light stimulates a biological motor neuron that has photosensitive protein expression, and an action potential is generated. (B,C) A chemical synapse of a neuromuscular junction transmits the potentials to a muscle fibre, (D) which causes the muscle to contract. Analogously, (E–I) in an organic artificial system, (E) light triggers a photodetector to generate output voltage spikes. (H–G) The voltage spikes produce electrical postsynaptic signals from an s‐ONWST to activate an artificial muscle actuator, (I) which causes the artificial muscle to contract. (F) Optical wireless communication via an organic optoelectronic synapse, with the patterned light signals representing the International Morse code of “ABC.” Reproduced under the terms of the Creative Commons Attribution‐NonCommercial license.^[^ ^110^ ^]^ Copyright 2018, The Authors, published by AAAS.

For example, engineering the OSC layer's microstructures in IGOSTs enables one to alter the kinetics of ion doping and de‐doping to control the decay time. The microstructures may be changed by varying the manufacturing parameters, such as the annealing temperature, or by utilizing a self‐assembled monolayer on substrates^[^ ^108^, ^109^ ^]^ (Figure 6). Notably, wearable smart devices and neural prostheses can only be created if artificial synapses and neurons can communicate with human beings. Hence, when devising bio‐hybrid neuromorphic systems, careful attention needs to be paid to the interplay between electrical components and biological systems like the integumentary system, cardiovascular system, neural network, and even individual neurons. IGOSTs have facilitated the development of organic sensing and motor neurons. These artificial neurons utilize the information received from sensory organs and exhibit motor responses. In recent studies, a photodetector, an IGOST, and a polymer actuator were employed to emulate a photosensitive neuron, a neuromuscular junction, and a muscle fibre, respectively, thus functioning as a synthetic sensory nerve^[^ ^110^ ^]^ (Figure 7). When exposed to light impulses, the photodetector creates excitatory presynaptic spikes. When presynaptic pulses are supplied to the electrolyte through the IGOST's gate electrode, excitatory postsynaptic currents are produced.

In 2016, Doyle and colleagues described a large‐scale, high‐density, organic material–based, conformable neural interface device (“NeuroGrid”) capable of simultaneously recording local field potentials (LFPs) and action potentials from the cortical surface.^[^ ^111^ ^]^ They demonstrated the feasibility and safety of intraoperative recording with NeuroGrids in anesthetized and awake subjects. Highly localized and propagating physiological and pathological LFP patterns were recorded, and correlated neural firing provided evidence about their local generation. Application of NeuroGrids to brain disorders, such as epilepsy, may improve diagnostic precision and therapeutic outcomes while reducing complications associated with invasive electrodes conventionally used to acquire high‐resolution and spiking data.^[^ ^111^ ^]^

Recently, significant attempts at system‐level integration of neuromorphic devices with biological components have linked artificial sensory nerves to biological cells or nerves. In these bio‐hybrid neuromorphic systems, artificial sensory nerves with sensing and information‐processing characteristics were built and employed.^[^ ^112^ ^]^ A bio‐hybrid neuromuscular junction was demonstrated by connecting a bimodal artificial sensory neuron to skeletal myotubes. When interacting with one's surroundings, sensory information is received from many types of receptors and processed to obtain an accurate and reliable perception of the environment. To mimic this complex perceptual behaviour, the devices used a pressure sensor and a photosensor to translate external haptic and visual input to electrical impulses. These signals were transferred by an ionic cable to an OST for signal integration and processing utilizing an indium‐tin‐oxide channel layer and polyvinyl alcohol (PVA) ion‐gel. This sensory neuron with haptic‐visual fusion displayed a variety of current levels based on the degree of synchronization between two sensory inputs, and this synchronization controlled the output voltage that stimulated the myotubes. Myoblast contraction became stronger as synchronization improved. As a result, this bio‐hybrid system successfully reproduced biological multimodal perception and body‐motion control.^[^ ^112^ ^]^

Similarly, Wang et al. created a flexible, conformal organic synaptic transistor in 2019 using extremely ductile Au electrodes and organic materials with low Young's modulus, such as DNTT semiconductor, organosilicon dielectric, and PDMS support.^[^ ^113^ ^]^ Such an organic synaptic transistor demonstrated silk‐like flexibility and conformability to 3D curved surfaces such as the human brain, coccinellidae, grasshoppers, and 4–8 mm hemispheres. More notably, the synaptic transistor could still function normally even when adhering to curved surfaces, including potentiation/depression characteristics, STP/LTP, and spike voltage‐dependent plasticity. The synaptic transistor may also function successfully after peeling, folding, and repeatedly glued on a 4 mm hemisphere surface. The unique approach of KPFM was utilized to illustrate the time‐dependent variation of surface potential difference between polarized and unpolarized areas in organosilicon dielectrics. These KPFM observations provided a direct observation of the delayed polarization process of the hydroxyl dipoles in dielectric, which is responsible for organic transistor synaptic characteristics, as well as the observed sweeping rate and sweeping range‐dependent hysteresis. The findings demonstrated the availability and benefits of organic transistors for realizing the flexible, conformal bionic synapse for prospective applications such as synaptic electronics, the intellectualized brain, and conformable smart tags.^[^ ^113^ ^]^

Organic synaptic transistors have enormous potential uses in therapeutic therapy. As we have already mentioned, one promising avenue is the development of neuroprosthetic devices.^[^ ^114^ ^]^ Combining organic synaptic transistors with neural interfaces makes it feasible to bridge the gap between damaged brain circuits and external devices, giving hope to people suffering from neurological illnesses or spinal cord injuries.^[^ ^115^ ^]^ These devices can potentially recover lost functions by enabling communication between artificial and organic brain systems.^[^ ^115^ ^]^ Furthermore, organic synaptic transistors show potential in neuromodulation.^[^ ^116^ ^]^ These devices might be used to produce individualized therapeutics for neurological disorders, including epilepsy, Parkinson's disease, and depression, by exploiting their capacity to modify synaptic strength. Controlling synaptic plasticity precisely might enable personalized therapies that adapt to an individual's particular demands, improving therapeutic outcomes. Organic synaptic transistors have the potential to transform artificial intelligence as well.^[^ ^117^, ^118^ ^]^ Traditional computing systems have difficulty replicating the brain's efficiency and agility. However, by utilizing the synaptic‐like behaviour of organic transistors, neuromorphic systems that closely resemble the neural networks of the brain may be developed. These organic synaptic‐based artificial neural networks offer the benefit of more effective processing and learning from complicated real‐world input.^[^ ^119^ ^]^ Their synaptic plasticity and flexibility allow them to learn from experience, make context‐based judgments, and deal with ambiguity. This paves the door for advanced applications in robotics, autonomous systems, and natural language processing by pushing the boundaries of machine learning, cognitive computing, and pattern recognition.

It is essential to highlight that implantable organic synaptic transistors are currently in the experimental phase, primarily focusing on proof‐of‐concept demonstrations and enhancing their performance. Scientists and engineers are actively exploring various materials and production methods to enhance the functionality, stability, and biocompatibility of these devices. Despite the intriguing potential of implantable organic synaptic transistors, several challenges need to be addressed before their widespread adoption becomes feasible. Ensuring the long‐term stability and reliability of these devices within the complex and dynamic environment of the human body is a significant hurdle. Implantable devices must demonstrate sustained functionality and performance over extended periods without degradation or damage.

Achieving biocompatibility is a critical consideration in the design of implantable devices. The organic materials employed in implantable organic synaptic transistors must be non‐toxic, non‐inflammatory, and compatible with biological systems. It is essential to minimize the immune response and the risk of rejection by the body. Integrating implantable organic synaptic transistors with existing brain circuitry and systems presents another formidable challenge. Developing scalable manufacturing techniques to produce large arrays of devices with high precision and reliability is crucial for practical implementation. Seamless integration of these transistors into neural circuits is essential for their effective functioning.

In conclusion, while implantable organic synaptic transistors hold great promise, they are still in the experimental stage, and research efforts are focused on improving their performance and addressing various challenges. Ensuring long‐term stability, achieving biocompatibility, and enabling seamless integration with existing brain systems are key areas of concern. Advancements in these areas will pave the way for the practical application of implantable organic synaptic transistors in diverse biomedical contexts.

### 2.3 Application to the brain as a neural link

Recent research on developing organic synaptic transistors has mainly concentrated on using artificial brains as neural links to simulate biological activities. A synthetic organic synapse imitates brain plasticity with simple architecture, inexpensive production, and minimal power requirements. Furthermore, the von Neumann bottleneck, a limitation in traditional computer architecture that restricts the overall performance of computing systems, arises due to the sequential nature of the von Neumann architecture, which is the predominant design used in most modern computers. Therefore, the von Neumann bottleneck is anticipated to be removed by intelligent computing systems that resemble human brains. Thus, the formation of neuro‐morphological systems is dependent on the growth of organic synapses. This section summarizes the advancements in organic‐synaptic‐transistor‐based brain systems.

**FIGURE 8:**
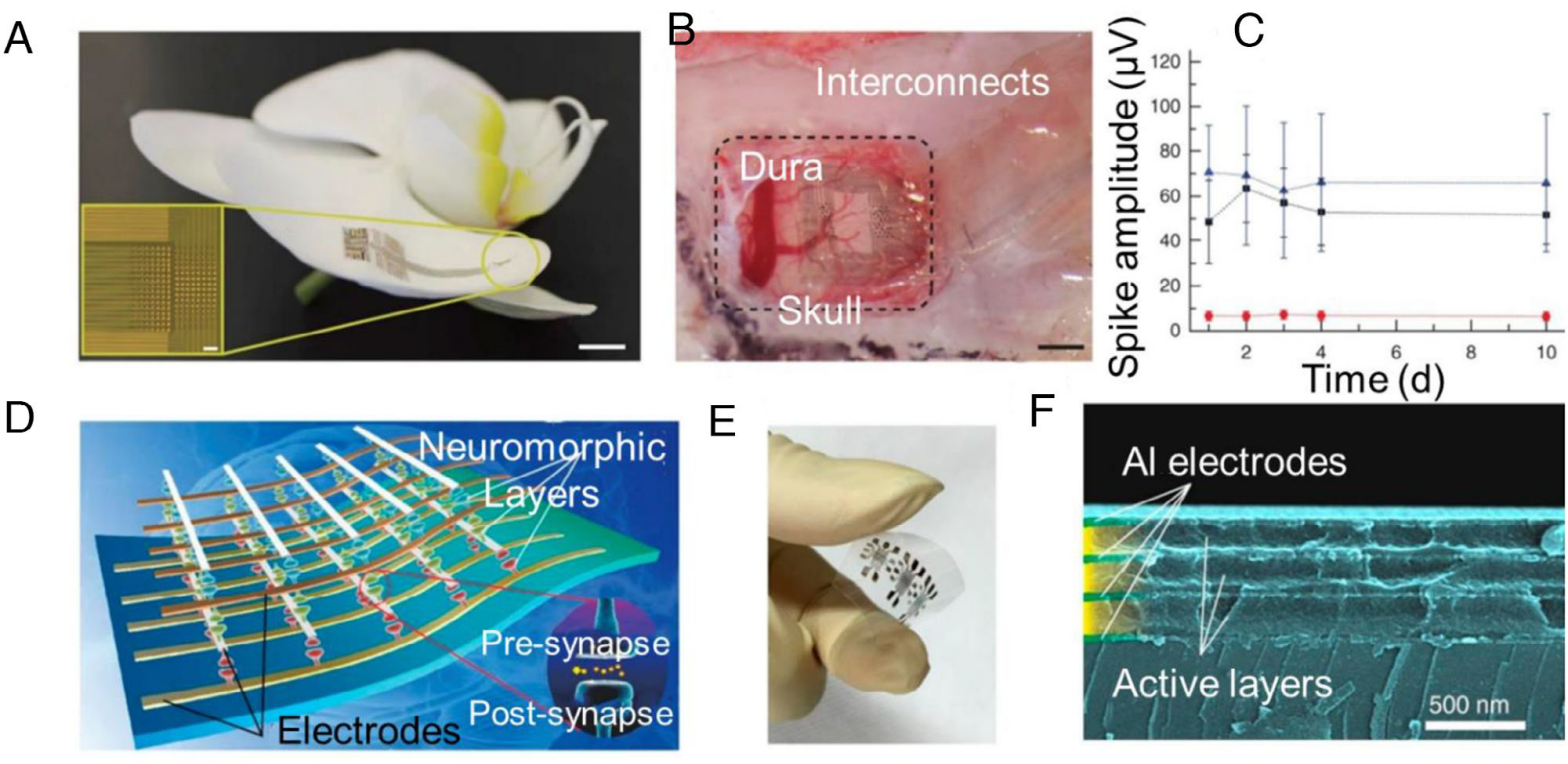
Application to the brain as a neural link. (A) The NeuroGrid conforms to the surface of an orchid petal (scale bar, 5 mm). Inset, optical micrograph of a 256‐electrode NeuroGrid (scale bar, 100 μm) (The size of the electrodes is 10 × 10 μm^2^, with an interelectrode spacing of 30 μm), (B) the NeuroGrid conforms to the surface of the rat somatosensory cortex (the edge of the resected dura is visible at the top left of the craniotomy (scale bar, 1 mm)), (C) mean and SD of the amplitude of the detected action potential waveforms across 10 d of recording (The average amplitude and variability of hippocampal waveforms (blue) are larger than those of cortical waveforms (black), The red curve demonstrates the spike detection threshold (rms. noise = 8 μV at 0.1–7500 Hz)). Reproduced with permission.^[^ ^20^ ^]^ Copyright 2014, Springer Nature Publishing AG; device structure and electrical behaviours of 3D‐ASN: (D) illustration, (E) photograph, and (F) cross‐sectional SEM image of the flexible 3D‐ASN based on e‐synapses. The active neuromorphic memory layer is sandwiched between the top and bottom electrodes, thus forming an e‐synapse. The top and bottom electrodes correspond to the presynaptic and postsynaptic neurons, respectively. Reproduced under the terms of the Creative Commons Attribution 4.0 International license.^[^ ^121^ ^]^ Copyright 2017, The Authors, published by Springer Nature.

Biological brain systems receive spatiotemporal patterns of action potentials from each external sensory stimulus, which are then processed. Numerous presynaptic neurons' relative timing of synaptic spikes reflects the characteristics of the stimulus. A fundamental requirement in cortical information processing activities is the distinction of various spatiotemporal input sequences using fault‐tolerant computing methodologies while consuming power. In addition to possessing essential synaptic properties akin to neurons, an electret‐based organic transistor synapse can reproduce handwritten designs with an impressive recognition accuracy of 85.88%.^[^ ^120^ ^]^ Due to their expandability and compatibility with biological systems, organic materials can be incorporated into the outermost region of the rat cerebral cortex, facilitating the capture of neuronal action potentials within this layer (Figure 8A–C).^[^ ^20^ ^]^ This device may assist in capturing the intraoperatively local field potential controlled spiking activity during epilepsy surgery. These techniques can help us better understand how cerebral activity varies across time and place, which might aid in the early detection and treatment of brain diseases. High device density and flexibility in artificial synaptic systems may arise from the development of 3D electronic devices that resemble synaptic networks. Yang's team created 3D artificial chemical synapse networks (3D‐ASN) using Cu‐doped poly (methyl silsesquioxane). An STDP learning rule, LTP/STP, PPF, and low power consumption (in the picojoule range for one spike) are just a few examples of the biological synaptic characteristics that this 3D‐ASN may emulate. To reduce the impact of crosstalk, 3D‐ANS's rectification characteristics can be utilized (Figure 8D–F).^[^ ^121^ ^]^

**FIGURE 9:**
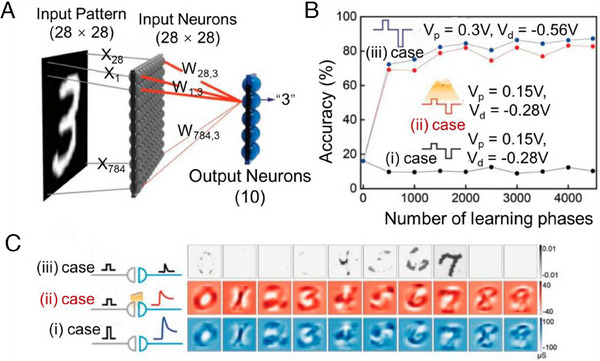
Application to the brain as a neural link. (A) Constituents of a single‐layer network for the “3” pattern recognition process. Input neurons (gray) and output neurons (blue) are fully connected by the individual synaptic weight (*w_i,j_*) (red line). Note that each input neuron is individually connected to each point of the input pattern. (B) Estimated recognition accuracy for the MNIST patterns as a function of the number of learning phases for three cases (i, ii, and iii). In case i), *V_p_* = 0.15 V and *V_d_* = −0.28 V are applied without light; in case ii), *V_p_* = 0.15 V and *V_d_* = −0.28 V are applied with light of *I_L_* = 8.16 mW cm^‐^^2^; and in case iii), *V_p_* = 0.3 V and *V_d_* = −0.56 V are applied without light. The initial power consumption of the potentiation (*P_p_*) is estimated for the three cases. (C) Reshaped 28 × 28 contour images of the final synaptic weights corresponding to the MNIST patterns after 4500 learning phases for the three cases. Reproduced with permission.^[^ ^123^ ^]^ Copyright 2019, Wiley.

The retina is a light‐sensitive layer that turns visual impulses into electrical pulses, allowing light‐controlled neurotransmitter release from different brain sites to modulate brain activity. Optical systems are distinctive components of biological neural links,^[^ ^122^ ^]^ and thus, many researchers have focused on synthetic optoelectronic synapses. For instance, using an organo‐lead‐halide‐perovskite, Wang's team developed two‐terminal optoelectronic synapses (Figure 9A–C).^[^ ^123^ ^]^ Han and colleagues successfully replicated essential optical synaptic behaviour by employing organic OTFTs based on the heterojunction of QDs and pentacene. The authors also used in situ Kelvin probe force microscopy to visually depict the dynamic carrier distribution process in illuminated and non‐illuminated conditions.^[^ ^124^ ^]^

Neuromorphic computing can also take advantage of organic photoreceptor synapses. Alpha Zero defeated the world chess champion using artificial intelligence techniques like self‐learning and neuromorphic computing. Alpha Go, for example, dominates the Go game.^[^ ^125^ ^]^ In recent years, the von neuromorphic computation‐based learning method for artificial neural networks has rapidly advanced^[^ ^125^ ^]^. In their study, Qian and colleagues utilized a heterojunction configuration involving copper‐phthalocyanine (CuPc) and p‐hexaphenyl (p‐6P). To build an artificial photoelectric synapse. The channel conductivity was thought to represent the synaptic weight that needed to be changed by introducing optical and electrical signals. The CuPc thin film underneath the p‐6P layer enhanced the morphology because of its unusual development pattern. In the Modified National Institute of Standards and Technology digital pattern training/recognition challenge, this artificial visual synaptic transistor had the highest recognition rate of 78%. It is based on a single‐layer perceptron artificial neural network. Our research, therefore, laid the experimental groundwork for the potential employment of optoelectronic synaptic transistors in high‐end artificial retinal neural networks in the future.^[^ ^126^ ^]^

**FIGURE 10:**
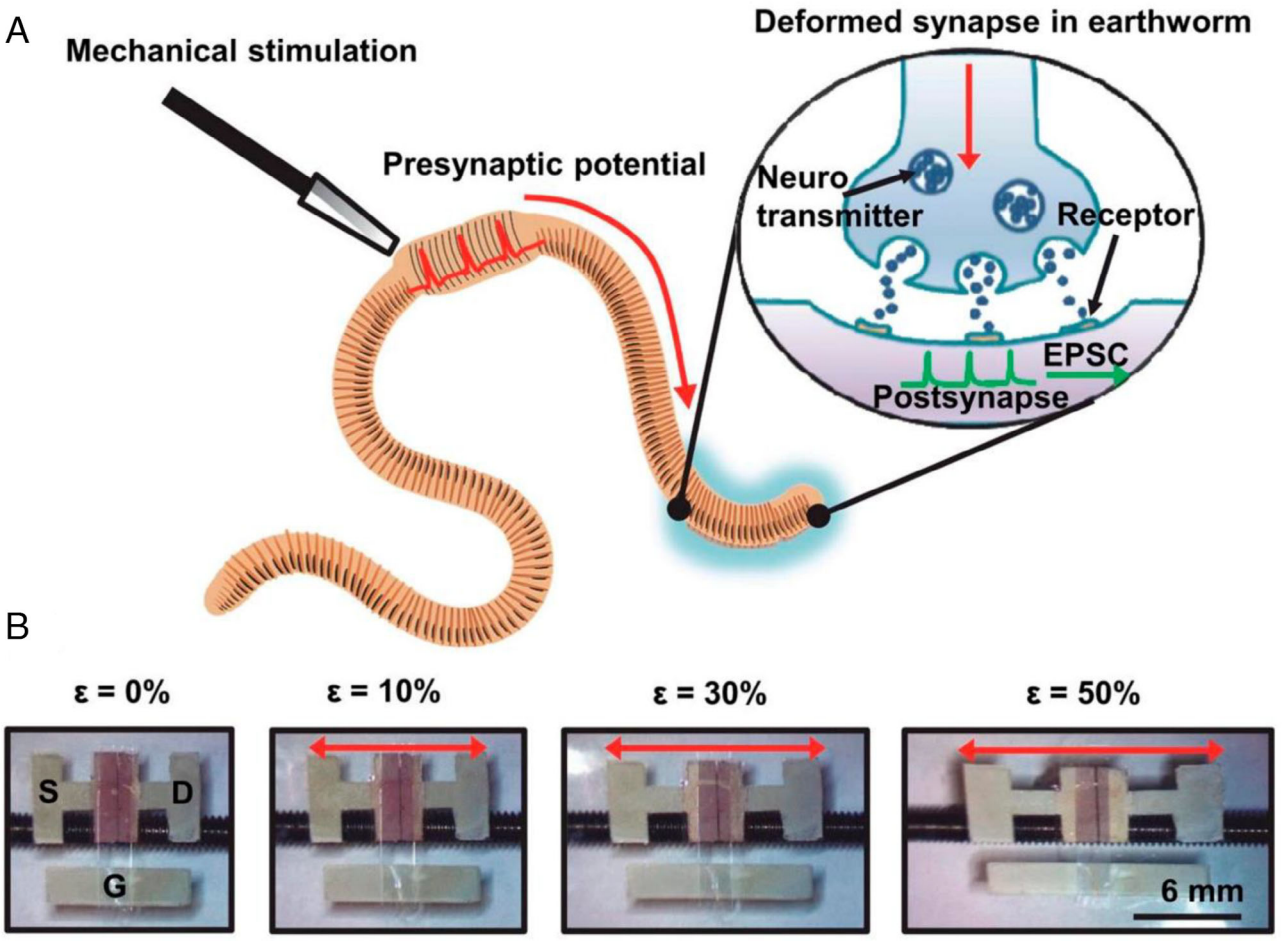
Application to the brain as a neural link. (A) Schematic of the deformable synapse and its synaptic transmission process in an earthworm (nerve impulses are created in the presynaptic neuron as a result of mechanical stimulation, and subsequently, ion channels in the postsynapse begin to open. The neurotransmitter‐containing vesicles travel toward the axon's end, freeing the neurotransmitters to flow through the synaptic cleft). (B) Optical images of the fully rubbery synaptic transistor under different levels (0%, 10%, 30%, and 50%) of mechanical strain (The P3HT‐NFs/PDMS layer has a thickness of ≈280 nm. The length and breadth of the channel are 60 m and 3 mm, respectively). Reproduced under the terms of the Creative Commons Attribution‐NonCommercial license.^[^ ^128^ ^]^ Copyright 2019, The Authors, published by AAAS.

Recent developments in artificial tactile systems include multifunctional prosthetics inspired by human skin and composed of flexible electrical components.^[^ ^127^ ^]^ Given the flexibility of organic materials, researchers have widely used them for developing flexible sensors. Yu et al., for example, created a soft neuro robot with organic synaptic transistors that can move by tapping an electronic skin (Figure 10A,B).^[^ ^128^ ^]^ Figure 10A depicts a schematic representation of an earthworm's elastic and malleable synapse and synaptic transmission mechanism. Nerve impulses are created in the presynaptic neuron as a result of mechanical stimulation, and subsequently, ion channels in the postsynapse begin to open. The neurotransmitter‐containing vesicles travel toward the axon's end, freeing the neurotransmitters to flow through the synaptic cleft. The neurotransmitters interact with the receptors on the postsynaptic neuron, allowing ions to flow across the membrane and create postsynaptic potential. Figure 10B illustrates a series of optical pictures of a completely rubbery synaptic transistor before and after uniaxial stretching by 10%, 30%, and 50% along the channel length direction. The P3HT‐NFs/PDMS layer has a thickness of ≈280 nm. The length and breadth of the channel are 60 m and 3 mm, respectively.

A biomimetic dual‐organic‐transistor‐based tactile perception system was demonstrated by Zhu's group. This system combines sensing transistors with organic synaptic transistors.^[^ ^129^ ^]^ Kim and colleagues built a biomimetic touch neuron with capacitive pressure sensors, ring oscillators, and a synaptic transistor.^[^ ^30^ ^]^ The ring oscillators converted the pressure sensor signal into a pulse electric signal to emulate the action potentials' method of biological signal transmission. As a result, the EDL transistors continuously increased the postsynaptic current. Lastly, a synthetic tactile neural system linked to a cockroach leg's biological afferent neurological system activated the reflex arc of the biological nerve. The discoveries discussed above demonstrated the numerous uses of artificial tactile systems in neural prostheses and neurorobotics. Chen and colleagues demonstrated the capacity of an additional artificial tactile nerve system to learn perceptual information by imitating sensory neurons. Flexible ionic cables, synaptic devices, and resistive tactile sensors were used to mimic the axon of transmission, the synapse, and the receptor of sensations to model how a sensory neuron processes information. The tactile sensor uses a pair of interfacial ions and electrons to convert pressure sensations into electrical impulses, which it then sent to the organic synaptic transistor via a flexible ionic wire. The organic synaptic transistor might also be activated by external pressure using the configuration's event‐activation capabilities.^[^ ^130^ ^]^ In conclusion, artificial tactile synaptic devices convert pressure inputs into electrical signals using capacitive or resistive sensors, which are then stored using synaptic transistors. Organic materials are likely to be utilized to replicate human touch or create prosthetic limbs due to their soft mechanical qualities and flexibility.

The current state of the field indicates that significant progress has been made; however, it is important to acknowledge that the technology is still in its early stages, and several challenges must be overcome before practical implementation can be realized. Further research and development are required to tackle the following challenges: One crucial challenge is ensuring the biocompatibility of implantable synaptic transistors for successful integration into the brain. It is imperative to use non‐toxic, non‐inflammatory materials compatible with neural tissue to minimize the risk of immune responses or tissue damage. Thorough investigations into the long‐term biocompatibility and stability of these devices within the brain environment are necessary. Establishing a reliable and efficient interface between the implantable synaptic transistors and neural tissue poses a significant hurdle. The electrodes or other sensing components of the devices must be designed to accurately capture and interpret neural signals while minimizing interference and signal degradation. Improving the signal‐to‐noise ratio and achieving high spatial and temporal resolution are critical factors to ensure effective neural communication. The long‐term stability and reliability of implantable synaptic transistors are vital for their practical application. These devices must maintain their functionality and performance over extended periods without experiencing degradation or damage. Factors such as device packaging, encapsulation, and protection against biofouling or degradation should be carefully addressed to ensure long‐term reliability.

## 3 CONCLUSION AND OUTLOOK

This review highlights recent advancements in applying organic synaptic transistors as implantable neural links. The forms and functions of organic synaptic transistors have recently undergone rapid improvements. Such multiterminal neural transistors have significant presynaptic inputs, enabling simultaneous signal processing. Consequently, the processing efficacy is greatly improved, and many critical synaptic processes can be replicated. Also, electrical stimulation primarily mimics the synaptic activity of most artificial neural devices.

Creating neuromorphic systems replicating biological sensory neurons (such as auditory, olfactory, and motor neurons) can further research environmental sensing, perception processing, and movement control for system collaboration. Despite the development of organic synapse materials and transistors, several challenges remain to be overcome. Understanding the functioning mechanisms of biological entities remains limited. For instance, the creation and communication mechanisms of organic synapses are largely unknown. Compared to most conventional semiconductor materials, OSC materials exhibit weaker mobility. To the facilitate the application of OSCs, their mobility should be enhanced through unique experimental techniques.

Furthermore, the stability of the existing organic synapse devices is low, as their performance is affected by ambient water and oxygen. These devices must work and perform over lengthy periods of time without degrading or being damaged. Factors such as device packing, encapsulation, and protection against biofouling or degradation should be carefully considered to guarantee long‐term dependability. Moreover, bundling of the corresponding devices can enhance their reliability and promote the development of adaptable, large‐scale artificial synaptic systems.

Organic, ecologically benign, and biodegradable materials can be used in artificial synaptic devices. Biocompatible organic synaptic devices can be used to develop embedded chips for supplemental medical care. Given the promising performance of single‐function artificial neural systems, researchers can aim to combine different neural networks to prepare hybrid platforms and exploit their advantages. Additionally, stretchable synaptic systems and high‐performance flexible and soft synaptic transistors can be built using artificial synapses. Integrated organic synaptic systems that can be printed, modified, and scaled up might be effective building blocks for future artificial synapses. Overall, organic synaptic transistor applications in biomedicine have enormous promise for transforming the diagnosis, treatment, and understanding of neurological illnesses. We can expect significant advancements in neuroprosthetics, brain‐machine interfaces, neurological research, and drug discovery as research advances and technology improves, ultimately leading to improved patient care and quality of life for people with neurological conditions.

## AUTHOR CONTRIBUTIONS

Y. K., H.K., and Y. Z. conceived and designed the work; S. B., and H. J. wrote the orginial manuscript; S. B., H. J., Y. L., and H. C., Y. K., H. K., and Y. Z. revised the manuscript.

## CONFLICT OF INTEREST STATEMENT

The authors declare no conflicts of interest.
